# The Mediating Effect of Social Support of Older Korean Adults’ Volunteering on the Relational and Life Satisfaction

**DOI:** 10.1093/geroni/igab046.2591

**Published:** 2021-12-17

**Authors:** Meeryoung Kim

**Affiliations:** Daegu University, Daegu, Kyongsang-bukto, Republic of Korea

## Abstract

For older adults wanting to maintain good health and stay active after retirement, volunteering is an important activity. Social capital is important factor for volunteering. Social support as a social capital, is a contributing factor that is important and needed by older adults who volunteer. Also as a result from volunteering, older adults can increase their social support through volunteering. This study examined whether emotional and instrumental social support mediate volunteering on both relational and life satisfaction. This study used the 6th additional wave of the Korean Retirement and Income Study (2016). Subjects for this study are over 60 years old and the sample size is 280. For data analysis Baron and Kenny's triangular regression analysis and the Sobel test were used for data analysis. Demographic variables were controlled. Volunteer variables such as volunteering asked by others or self-motivated, whether only one type of volunteering or more, professional volunteering, and volunteer hours were used as independent variables. Emotional and instrumental social support were used as mediators. Relationship satisfaction and life satisfaction variables were used as dependent variables. Emotional and instrumental support partially mediate volunteering asked by others to influence relational and life satisfaction. In addition, emotional support and instrumental support mediate “more than one kind of volunteering” to influence relationship satisfaction. As such, emotional and instrumental support through volunteering has a mediating effect on relationship satisfaction and life satisfaction.

